# Self-assembling HA/PEI/dsRNA-p21 ternary complexes for CD44 mediated small active RNA delivery to colorectal cancer

**DOI:** 10.1080/10717544.2017.1386732

**Published:** 2017-10-10

**Authors:** Chen-Lin Feng, Yan-Xing Han, Hui-Hui Guo, Xiao-Lei Ma, Zhi-Qiang Wang, Lu-Lu Wang, Wen-Sheng Zheng, Jian-Dong Jiang

**Affiliations:** State Key Laboratory of Bioactive Substance and Function of Natural Medicines, Institute of Materia Medica, Chinese Academy of Medical Sciences and Peking Union Medical College, Beijing, PR China

**Keywords:** Colorectal cancer, ternary complexes, small active RNA, hyaluronic acid, tumor targeted

## Abstract

Our previous work proved that sequence specific double strand RNA (dsRNA-p21) effectively activated p21 gene expression of colorectal cancer (CRC) cells and consequently suppressed CRC growth. However, efficient delivery system is a significant challenge to achieve sufficient therapy. In this study, a self-assembled HA/PEI/dsRNA-p21 ternary complex (TC-dsRNA-p21) was developed for the tumor-target delivery of dsRNA-p21 into CRC cells. Hyaluronic acid (HA) was introduced to shield the PEI/dsRNA-p21 binary complexes (BC-dsRNA-p21) for reducing the cytotoxicity of PEI and for increasing the tumor-targeted intracellular uptake by cancer cells through HA-CD44 mediated endocytosis. Comparing to the BC-dsRNA-p21, the TC-dsRNA-p21 showed increase in size, decrease in zeta potential, low cytotoxicity as well as high stability in physiological conditions due to the anionic shielding. Confocal microscopy analysis and flow cytometry confirmed that TC-dsRNA-p21 had high transfection efficiency in the CD44-abundant Lovo cells, as compared with binary complex. *In vitro* physiological experiment showed that, comparing to the control group, the TC-dsRNA-p21 effectively activated the expression of p21 mRNA and P21 protein, causing blockage of cell cycle at *G*_0_/*G*_1_ phase and suppression of cancer cell proliferation as well as colony formation. Furthermore, *in vivo* distribution experiment demonstrated that the TC-dsRNA-p21 could effectively accumulate at rectal wall for up to 10 h, following *in situ* application. These findings indicated that TC-dsRNA-p21 might hold great potential for delivering dsRNA-p21 to treat CRC.

## 1. Introduction

Colorectal cancer (CRC) is the third most commonly diagnosed cancer and the third leading cause of cancer death worldwide (Siegel et al., 2017). Conventional cytotoxic chemotherapy such as 5-fluorouracil based adjuvant remains to be the first-line treatment. Severe side effect coupled with high incidents of drug resistance is still the main challenge. It’s urgently desirable to find an alternative way for the treatment of CRC (Wang et al., 2017). RNA-induced gene activation (RNAa) is a new mechanism to inhibit cancer growth by augmenting the expression of tumor suppressing genes (Zheng et al., 2014). Our previous work proved that the sequence specific double strand RNA: dsRNA-p21 could successfully hinder CRC growth via stimulating p21 gene expression. However, the difficulty of delivering these therapeutic molecules limited their further clinical application (He et al., 2013).

Small RNAs are unstable, relatively high molecular weight and negative charged molecules, which are impermeable to the cellular membrane (Chen et al., 2016). To address these issues, a series of non-viral gene delivery systems, including cationic lipids and polymers, have been concentrated due to the advantages such as low immunogenicity, ease of manufacturing, stability and high flexibility regarding the size of the trans-gene delivered (Aldawsari et al., 2011). Among which, polyethyleneimine (PEI) is the most valuable cationic polymer for nucleic acid transfection (Thomas & Klibanov, 2002; Fischer et al., 2003). PEI can effectively condense RNA to form tight complexes and protect the payload from degradation by RNase. The positive-charged complexes can bind to negative-charged cell membrane and exhibit considerable transfection efficiency (Seib et al., 2007). Besides, PEI could facilitate nucleic acids to escape from lysosomes, through the protonation of amines in the PEI molecule, which is called proton sponge effect (Behr, 1997; Akinc et al., 2005). However, high toxicity and the aggregation nature in the physical conditions plagued its application *in vivo* (Chollet et al., 2002; Wang et al., 2008). To avoid such adverse effects, several strategies have been explored to reduce the cytotoxicity, increase the stability in physical condition and improve the transfection ability of PEI/RNA complexes, such as shielding with anionic polymers (Jeon et al., 2008; Kurosaki et al., 2009), chitosan (Jiang et al., 2008) or liposome (Schafer et al., 2010).

In the current study, we developed electrostatically self-assembled ternary complexes as dsRNA-p21 carrier to treat CRC. The ternary complexes incorporate hyaluronic acid (HA) with branched-PEI. HA, the major constituent of the extracellular matrix (ECM), is a natural linear polysaccharide with alternating disaccharide units of B-1,3-*N*-acetylglucosamine linked to B-1,4-glucuronic acid (Sahiner et al., 2017). HA has been broadly used in tissue engineering and drug delivery for its excellent biocompatibility and biodegradability (Mironov et al., 2005; Lee et al., 2007). Very interestingly, HA is the specific ligand for CD_44_ (Underhill, 1992), which are highly expressed in many tumor cells, including CRC (Haruyama et al., 1999; Zalewski et al., 2001). HA-modified nanoparticles were reported to possess capability of assisting tumor targeting and drug internalization via CD44 receptor-mediated endocytosis (Almalik et al., 2013; Li et al., 2014). Furthermore, HA, which was full of hydroxyl groups, could exert bio-adhesive properties by forming solid hydrogen bonds with oligosaccharide chains on the mucous surface and increase drug retention at the lesion site (Yamamoto et al., 2000; Chiou et al., 2009). Nanoparticles covalently anchored with HA was reported to act as site-adherent and site-retained depots under *in vivo* conditions, which was critical for topical administration (Margalit et al., 1992; Yerushalmi et al., 1994). We hypothesized that the combination of HA shielding and PEI condensation would further enhance the efficiency in tumor-targeted dsRNA-p21 transfection, reduce the toxicity of PEI/dsRNA-p21 complex, improved the stability in physical condition, as well as increase the retention of active reagent on the lesion site. The stability, cytotoxicity, cell transfection, *in vitro* physiological effect and *in vivo* drug distribution for the designed HA/PEI/dsRNA-p21 ternary complexes were systematically investigated in this study.

## 2. Materials and methods

### 2.1. Materials

#### 2.1.1. Chemicals

Sodium (HA, molecular weights 100 kDa) was purchased from Tian-sheng Bio-Tech Co., Ltd (Shandong, China). Branched polyethyleneimine (PEI) with the MW of 10,000 were purchased from Aladdin Reagent Database Inc. (Shanghai, China). The dsRNA-p21 sense and antisense were designed as described by Li et al. (2014) and chemically synthesized by Gene-Pharma (Suzhou, China). PE-dsRNA-p21 (the 5′-end of the sense strand in dsRNA-p21 was conjugated with PE dye), FITC-dsRNA-p21 (the 5′-end of the sense strand in dsRNA-p21 was conjugated with FITC dye), DAPI, Lyso Tracker™ Red, Hoechst 33342 were purchased from Thermo-fisher Inc. (Thermo-fisher, Waltham, MA). P21 primary antibodies, β-actin primary antibodies, horseradish peroxidase-conjugated secondary antibodies were obtained from Seajet Scientific Inc. (Beijing, China). DMEM medium containing 10% Heat inactivated Fetal Bovine Serum (FBS), 1% l-glutamine, 1% Penicillin (10,000 U/ml) + Streptomycin (10 mg/ml) + Nystatin (1250 U/ml) solution, 0.25% Trypsin-EDTA solution, 3-(4,5-dimethylthiazol-2-yl)-2,5-diphenyltetrazolium bromide (MTT) were from Thermo-fisher Inc. (Waltham, MA). The materials for the agarose electrophoresis gel, SYBR Green II RNA gel stain were purchased from Molecular Probes Inc. (Vienna, Austria) Triton^®^ X100 were purchased from Millipore Sigma Inc. (Darmstadt, Germany) All other chemicals were purchased from Fisher Scientific (Waltham, MA, USA) and used as received. All other reagents were of analytical grade. All water used in this study (ddH_2_O) was freshly double distilled.

#### 2.1.2. Reagent

All RNA sequences were chemically synthesized and purified by Thermo-fisher (Waltham, MA). The dsRNA-p21 composed of the sense strand 5-GGCUACGUCCAGGAGCGCACC-3 and the antisense strand 5-ΜGCGCUCCΜGGACGUAGCCUU-3; the dsRNA-p21 labeled at the 5-end of the sense strand with FITC (excitement 488, emission 525 nm) and labeled at the 5-end of the sense strand with PE were used as fluorescent dsRNA-p21 probes, respectively. The final concentration of the duplexes was 50 M in TE buffer.

#### 2.1.3 Cell culture

293TT and Lovo cells were abstained from China Infrastructure of Cell Line Resources (Beijing, China). Cells were cultured in DMEM medium (Thermo-fisher, Waltham, MA) supplemented with 10% (v/v) fetal bovine serum (FBS), penicillin (100 U/mL), and streptomycin (100 μg/mL). All cell lines were cultured in a humidified atmosphere containing 5% CO_2_ and maintained at 37 °C.

### 2.2. Methods

#### 2.2.1. Formation and characterization of HA–PEI–dsRNA-p21 ternary complexes (TC-dsRNA-p21)

##### 2.2.1.1. Preparation of PEI/dsRNA-p21 binary complexes (BC-dsRNA-p21)

The PEI/dsRNA-p21 binary complexes (BC-dsRNA-p21) were prepared at PEI/dsRNA-p21 ratios of 2:1 (w/w). The PEI 10,000 was capable of effectively condensing dsRNA-p21 to form stable complexes above a (w/w) ratio of 1.5. For the subsequent experiments, the complexes with the weight ratio (2:1) were used as representative samples. About 50 µg dsRNA-p21 was dissolved in 50 µl buffer (150 mM NaCl, 10 mM HEPES, pH 7.4), 100 µg PEI was dissolved in 50 µl of the same buffer in a separate vial. After 10 min incubation at room temperature, the PEI solution was then pipetted to the dsRNA-p21 solution, vortex and incubated for another 30 min at room temperature.

##### 2.2.1.2. Preparation of HA/PEI/dsRNA-p21 ternary complexes (TC-dsRNA-p21)

Due to the electrostatic attraction, HA was assembled to construct HA/PEI/dsRNA-p21 ternary complexes. As described in the previous research (Xu et al., 2009), 50 µg HA (100,000 Da) was dissolved in 50 µl buffer (150 mM NaCl, 10 mM HEPES, pH 7.4), the BC-dsRNA-p21 was added to HA solution to achieve an HA/PEI ratio (w/w) of 1–8 and incubated at room temperature for 10 min.

##### 2.2.1.3. Particle size, polydispersity index (PDI) and zeta potential test

The particle size, zeta potential and polydispersity index (PDI) of each of the drug-loaded sample were determined by dynamic light scattering (DLS) using Zeta sizer Nano (Malvern Instruments Ltd., Malvern, UK). Samples were diluted in buffer (150 mM NaCl, 10 mM HEPES, pH 7.4) to the dsRNA-p21 concentration of 5 µg/ml. The viscosity of 0.8872 and refractive index of 1.590 at 25 °C were chosen for analysis. Three measurements with 10 sub-runs were performed for each sample. Data were processed and were then expressed as mean ± SD.

##### 2.2.1.4. Morphology test

Transmission electron microscopy (TEM) was used to test the morphology. A drop of dsRNA-p21 encapsulated BC and TC was placed on a copper grid, respectively. Phosphotungstic acid was applied for 5 min as a negative stain. The solution that overflowed was cleared and dried in the air. The grid was then examined under a transmission electron microscope (Hitachi H-7650, Hitachi, Ltd., Tokyo, Japan).

##### 2.2.1.5. dsRNA-p21 condensation assay

Gel retardation assay was used to evaluate the dsRNA-p21 condensation ability of formulations. Samples containing 1 µg of dsRNA-p21 were mixed with the loading buffer (6×) and loaded in the well of 4% agarose gel in the presence of 1% SYBR Green II RNA gel stain (Molecular Probes, Eugene, OR). Samples were applied to 90 V electrodes in 0.5 × Tris-EDTA (TEA) (Millipore Sigma, Kenilworth, NJ) buffer for 20 min. RNA bands were tested with ChemiDoc XRS + electrophoretic imaging system (Bio-Rad Laboratories, Berkeley, CA, USA).

##### 2.2.1.6. Stability of complexes in the absence of RNase

In order to investigate the protection of formulations to dsRNA-p21, samples containing 30 µg of dsRNA-p21 were incubated with 20 μg/ml ribonuclease A (RNase A) at 37 °C for 1.5 h, free dsRNA-p21 was used as control. At the end of incubation, 1 ml of 0.25 M EDTA solution was added to inactivate the RNase and samples were disassembled by adding 60 µl of 20% SDS. About 4.0% agarose gel electrophoresis was conducted in the presence of 1% SYBR Green II RNA gel stain (Molecular Probes, Eugene, OR) to monitor the integrity ofp21-saRNA-322.

##### 2.2.1.7. Aggregation study

All complexes were prepared as mentioned above. Then the NaCl concentration was adjusted to 150 mM, which was comparable to the physiological salt condition. The samples were incubated for different period of time to measure the size by DLS.

#### 2.2.2. *In vitro* cytotoxicity

The 3-(4,5-dimethylthiazol-2-yl)-2,5-diphenyltetrazolium bromide (MTT) assay was performed to evaluated the cytotoxicity. Briefly, approximately 1 × 10^4^ Lovo cells or 1 × 10^4^ 293T were seeded in each well of a 96-well plate and cultured for overnight at 37 °C in a CO_2_ incubator. The medium was then replaced with 200 μL of fresh complete medium containing samples achieve dsRNA-p21 concentrations from 100 μg/ml to 1000 μg/ml. After a 48 h incubation, the medium was replaced with 200 μl of MTT (0.5 mg/ml), followed by incubation at 37 °C for additional 4 h. The medium was replaced with DMSO (200 µL) to dissolve the deposited for mazan crystals. The optical density (OD) of each well was determined on a spectrophotometer (Bio Tek Instruments, Inc., Winooski, VT, USA) with 570 nm as the test wave length. Each polymer concentration was tested in five times. The reduction in viability of each group was expressed as a percentage of the untreated cells, which were considered to be 100% viable. Each sample was tested in five well and all experiments were performed three times.

#### 2.2.3. Intracellular uptake study

Two methods flow cytometric and confocal laser scanning microscopy (CLSM) analysis were applied to investigate the cellular uptake of these carriers. The Lovo cells or HEK 293 T cells were cultured in 20 mm glass bottom cell culture dish (NEST Biotechnology, Wuxi, China) at 1 × 10^5^ cells/dish with DMEM medium containing 10% FBS for 24 h. The cells were treated with PE-dsRNA-p21 containing formulations for Lovo cells (at a concentration of 50 nM dsRNA-p21) and FITC-p21-saRNA containing formulations for 293 T cells (at a concentration of 50 nM FITC-p21-saRNA), respectively for 6 h at 37 °C in a CO_2_ incubator. Untreated cells were used as the control. The cells were washed twice using PBS. After being fixed in 3% paraformaldehyde solution, the cells were rinsed several times in PBS, permeated with 1% Triton ×100 and stained with DAPI. Cells were immediately visualized using CLSM *via* LSM710 (Zeiss, Oberkochen, Germany, excitation was by Argon laser at 488 nm, and emission was captured through a 518 nm long pass filter for PE; excitation at 358 nm, emission at 461 nm for DAPI; excitation at 488 nm, emission at 670 nm for PE).The magnification objective lens was 40×. For flow cytometric experiment, cells were harvest, rinsed by PBS twice, trypsinized with 0.25% trypsin-EDTA, and fixed in (80%) cold ethanol at 4 °C for 24 h. Fixed cells were collected by washing with PBS twice and centrifuged at 300*g* for 5 min. Cells were suspended and immediately analyzed at BD FACS Calibur (BD Biosciences, San Jose, CA, USA). About 10,000 events were collected and analyzed. Excitation was by a single 15 mW argon-ion laser beam (488 nm). Emission was collected through a 518 nm and 670 nm band pass filter. In all analyzes, cell debris and aggregates were excluded by setting a gate on the plot of side-scattered light (SSC) vs forward-scattered light (FSC). Non-treated cells were analyzed in parallel as a negative control.

#### 2.2.4. Inspiration effect of dsRNA-p21 containing systems on p21 gene

The gene inspiring efficiency of the formulations on Lovo cells were evaluated using RT-PCR and Western blotting method. The Lovo cells (2 × 10^5^ cells/well) were seeded on 12 well cell culture plate and treated with the free dsRNA-p21, dsRNA-p21 containing formulation (at a concentration of 50 nM dsRNA-p21). Untreated cells were used as the control.

##### 2.2.4.1. Real-time quantitative reverse-transcriptase polymerase chain reaction (RT-PCR)

About 48 h after treatment, cells were isolated from the cell culture plate using TRIzol^®^ Plus RNA Purification Kit (Invitrogen, Carlsbad, CA, USA) in accordance with the manufacturer’s instructions. The concentration of total RNA was measured by spectrophotometer ND2000 (Thermo-Scientific, Waltham, MA). About 5 ng of total RNA was used for quantitative RT-PCR, using Power SYBR^®^ Green RNA to-CT™ 1-Step Kit (Applied Biosystems, Foster City, CA, USA). PCR amplification was applied on the Applied Biosystems 7500 Fast Real-Time PCR System (Applied Biosystems, Foster City, CA, USA), including an initial denaturation step (95°Cfor 10 min), 40 cycles of denaturation (95 °C for 10 s), and annealing (60 °C for 1 min). Values are showed as fold-differences compared to that of untreated reference group. Expressions were normalized to glyceraldehyde-3-phosphate dehydrogenase (GAPDH). All experiments were done in triplicate and independently validated three times. The p21 PCR primer sequences: 5′-CTTCGACTTTGTCACCGAGA-3′ (forward), 5′-GGTCCACATGGTCTTCCTCT-3′ (reverse); GAPDH PCR primer sequences: 5′-AGAACATCATCCCTGCCTCT-3′ (forward), 5′-CTGCTTCACCACCTTCTTGA-3′ (reverse).

##### 2.2.4.2. Western blotting analysis

As described before, cells were collected 48 h after treatment, rinsed twice with ice-cold PBS, lysed on ice in Pierce RIPA buffer (Thermo-Scientific, Waltham, MA) for 30 min. Cell lysates were clarified by centrifugation at 16,000*g* for 20 min at 4 °C. Cell lysates (concentration of total protein was adjusted by adding certain amount of RIPA buffer) were subjected to 12% SDS-PAGE gels, separated by SDS-PAGE and electrophoretically transferred to PVDF membranes (Life technologies, Camarillo, CA, USA). Membranes were blocked with 5% skim milk and then incubated overnight with P21 primary antibodies (Seajet Scientific Inc., Beijing, China) followed by matching horseradish peroxidase-conjugated secondary antibodies. After washing each with TBST several times, immobilon Western Chemiluminescent HRP Substrate (Millipore, Kenilworth, NJ) was added and the result was tested with ChemiDoc XRS + electrophoretic imaging system (Bio-Rad Laboratories, Berkeley, CA, USA), expressions were normalized to β-actin.

#### 2.2.5. *In vitro* pharmacological effect

##### 2.2.5.1. Anti-proliferation activity

As described in our previous research (Wang et al., 2017), the cell count analysis using TC 20TM Automated Cell Counter (Bio Rad, Hercules, CA, USA) was performed to assess the cell proliferation. In brief, Lovo Cells were treated with different formulations containing 50 nM of dsRNA-p21 for 24 h untreated cells were used as references. Following treatments, cells were plated in 12-well plates at a density of 5 × 10^4^/ml. Every 24 h for the following six days, a batch of cells were collected and the cell number was detected. All experiments were performed in six duplicate.

##### 2.2.5.2. Cell cycle analysis

Flow cytometry assay was used to analyze cell cycle as described before (Wang et al., 2017). In brief, 48 h after treatment, cells were harvest, rinsed by ice-cold PBS twice and fixed in (80%) cold ethanol at −20 °C for 1 h. Fixed cells were collected by centrifuged at 500*g* for 5 min and washing with PBS twice. About 500 ml of podium iodide (PI) staining solution were added into each tube, incubated for 30 min at room temperature in the darkness. The stained cells were immediately analyzed at BD FACS Calibur (BD Biosciences, San Jose, CA, USA). Cell cycle was showed as the percentage of cells in *G*_0_/*G*_1,_ *G*_2_/M and S populations.

##### 2.2.5.3. Colony formation

As described in previous research, Lovo cells were transfected with TC-dsRNA-p21 containing 50 nM of dsRNA-p21 for 12 h, BC-dsRNA-p21 and scramble RNA treated cells at the same dsRNA-p21 concentration as well as the untreated cells were used as references. Following treatments, cells were transferred to six-well plates and seeded at a density of 1.0 × 10^3^cells per well and continued culturing for another 12 days. Colony formation was analyzed by staining cells with a 0.05% crystal violet solution for 1 h.

#### 2.2.6. *In vivo* retention and bio-distribution

The animal study was carried out according to the protocol approved by the Committee of the Ethics of Animal Experiments of the Chinese Academy of Medical Sciences and Peking Union Medical College. Eight-to Ten-week-old male BALB-c nude mice were purchased from Beijing Weitong Lihua Experimental Animal Technology Co. Ltd (Beijing, China). All animals in this study were housed under pathogen-free conditions and were maintained in accordance with the guidelines of the recommendations for the Care and Use of Laboratory Animals of the National Institutes of Health.

Living small animal imaging was applied to evaluate the *in vivo* retention and bio-distribution of delivery system. Prior to treatment, the mice were fasted for 6 h with free access to water. The cy-5-conjugated dsRNA-p21 was formulated in Ternary complexes, administered into the rectum 1 cm above the anus of nude mice using a gavage needle. The cy5-dsRNA-p21 water solution was administered as a reference. Five min and 3 h post administration, two-dimensional (2D) fluorescence signal (FRS) and 3D fluorescent tomography (FRT) were acquired of each mouse carcass using the IVIS Spectrum Imaging System. Mice were positioned individually on the imaging platform in the supine position. In each case, the 2D FRS was firstly performed followed immediately by 3D FRT without interval perturbation of the carcass. The tomography algorithm was available on the IVIS Spectrum for 3D FRT configuration, which produces the 3D location of the fluorescence source within the 3D surface of the study animal. About 10 h post administration, the mice were sacrificed. The colorectal tissue was prepared as fresh-frozen tissues, counterstained with DAPI (Thermo-fisher, Waltham, MA), and observed using fluorescent microscopy (×100; Carl Zeiss, Oberkochen, Germany).

#### 2.2.7. Rectal irritation

About 12 h post administration, the mice were sacrificed. The rectum was isolated and washed with a saline solution, fixed in 10% neutral carbonate buffered formaldehyde, embedded in paraffin and cut into slices. Slices were stained with hematoxylin-eosin and observed under light microscopy for morphology.

### Statistical analysis

Data are expressed as mean ± standard deviation (SD). Statistical analysis of data was done with Student’s *t*-test, using SPSS15.0 Sigma Plot software (St. Louis, MO, USA). The correlation between variables was analyzed using Pearson correlation coefficient and *p <* .05 was considered to be a statistically significant difference.

## 3. Results and discussion

### 3.1. The physiochemical properties of the BC-dsRNA-p21 and TC-dsRNA-p21

All of the complexes in this research were prepared in NaCl at concentration of 20 mM with pH value of 7.4. The BC-dsRNA-p21at PEI/dsRNA-p21 weight ratio of 2 and the HA was assembled to construct HA/PEI/dsRNA-p21 ternary complexes via electrostatic attraction.

#### 3.1.1. Particle size and zeta potential

**Figure 1.**
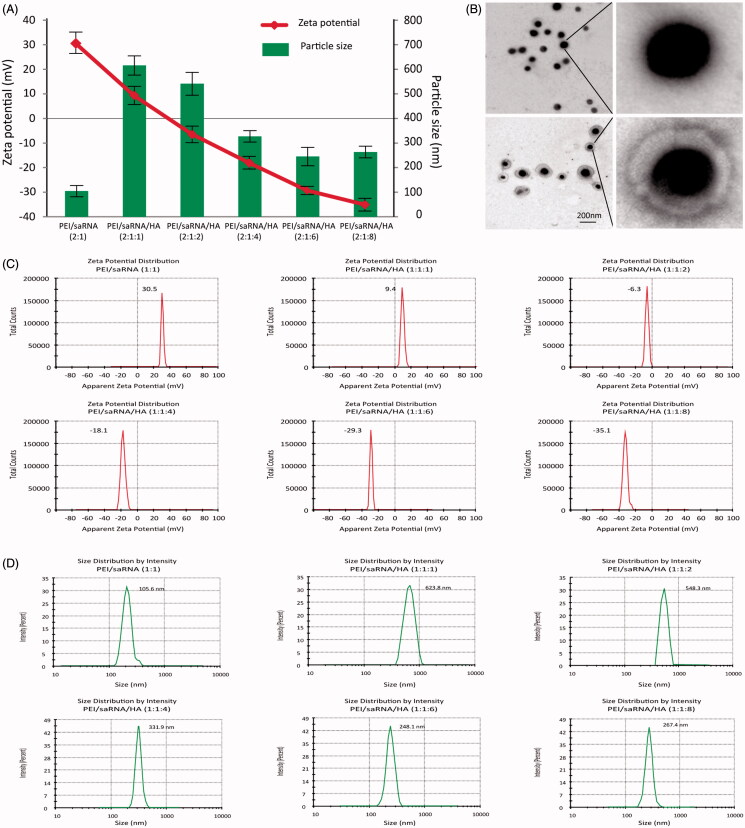
The mean diameter, ξ-potential and morphology of PEI/dsRNA-p21 binary complexes and various HA-PEI/dsRNA-p21 ternary complexes. The particle size and ξ-potential were determined using dynamic light scattering (DLS); morphology was tested using transmission electron microscopy (TEM) analyzes. (A) Mean diameter and ξ-potential. HA-PEI/dsRNA-p21 ternary complexes had HA/PEI ratios of 1:1, 1:2, 1:4, 1:6 and 1:8. Error bars represent mean ± SD for *n* = 5. (B) Morphology of PEI/dsRNA-p21 binary complexes and HA-PEI/dsRNA-p21 ternary complexes at HA/PEI ratio of 6. Scale bar, 100 nm. (C) ξ-Potential distribution. (D) Size distributions.

The particle size, PDI and zeta potential of binary and ternary complexes were determined with a Zeta sizer Nano ZS and the morphology was visualized through transmit emission microscopy. As shown in Figure 1, the mean sizes of the PEI/dsRNA-p21binary complex at PEI/dsRNA-p21 weight ratio of 2 were 106 ± 3.3 nm with the PDI of 0.22. Whereas, with the prolongation of incubation time, larger sizes with PDI up to 0.81 ± 0.10 were observed, indicating an aggregation. It is consistent with previous research that, PEI/RNA complexes display low colloidal stability and tend to aggregate in the presence of salts and serum proteins (Lungwitz et al., 2005; Neu et al., 2005). As shown in Figure 1(A and D), the particle size of ternary complexes was significantly enlarged when the HA was added to binary complexes. When the HA/PEI ratio was 1 and 2, complexes reached to 871.8 ± 11.47 nm and 734.5 ± 13.36 nm, respectively. The particle size was decreased as increasing the HA dosage. When the ratio of HA/PEI ratio reached to above 6, the particle size was about 260 ± 8.86 nm and stay invariable. This phenomenon could be explained by that addition of negative-charged HA electrostatically neutralized the charge dense of PEI/dsRNA-p21 complexes, which lead to the less compact structure with enlarged particle size. When the HA/PEI ratio reached to 1 or 2, the ξ-potential of ternary complexes was near neutral, causing chunky particle sizes. With further increment of HA dosage, the size of ternary complexes was reduced. The zeta potentials of binary complexes were +18.4 ± 1.1mv, showing that the particles were covered with excess positive-charged PEI. Increasing the dosage of HA, the ξ-potential of ternary complexes was decreased. When the HA/PEI ratio was 6, the ξ-potential reached − (29.1 ± 2.4) mv and kept invariable with increase of HA dosage (Figure 1(A and C)). The morphology image showed that, binary complexes were tight spheres, while the morphology of ternary complexes showed the structure of ternary complex with dark core of PEI/dsRNA-p21 and light gray shell of HA, which was consistent with DLS test (Figure 1(B)). All the results proved that, as adding to the binary complexes, HA was assembled outside the core and formed a negative corona.

#### 3.1.2. Condensation ability, stability and aggregation

To achieve optimal biological activity, the dsRNA-p21 should be in a good integrity before reaching the target cells; thus, RNA condensation, protecting capability against RNase and physiology stability of the BC-dsRNA-p21 and TC-dsRNA-p21 were evaluated.

**Figure 2.**
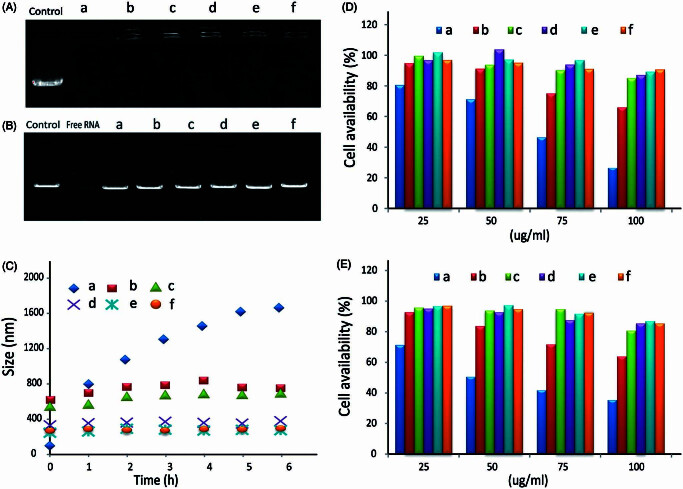
The gel retardation, cytotoxicity and stability assay of PEI/dsRNA-p21 binary complexes and various HA/PEI/dsRNA-p21 ternary complexes. (A) Condensation ability. (B) Protection capability against RNase. (C) The size of different complexes in physiological salt condition was determined by DLS. Error bars represent means ± SD for *n* = 5. (D) The effect of HA dosage on Lovo cell viability of different complexes were evaluated by MTT. (E) The effect of HA dosage on 293 T cell viability of different complexes were evaluated by MTT. Error bars represent means ± SD for *n* = 5. a represents PEI/dsRNA-p21 at weight ratio of 1:1; b represents HA/PEI/dsRNA-p21/at HA/PEI weight ratio of 1; c represents HA/PEI/dsRNA-p21/at HA/PEI weight ratio of 2; d represents HA/PEI/dsRNA-p21/at HA/PEI weight ratio of 4; e represents HA/PEI/dsRNA-p21/at HA/PEI weight ratio of 6, f represents HA/PEI/dsRNA-p21/at HA/PEI weight ratio of 8; and g represents HA/PEI/dsRNA-p21/at HA/PEI weight ratio of 10.

The gel retardation assay was used to evaluate dsRNA-p21 condensation ability. As shown in Figure 2(A), the band of free dsRNA-p21 did not appeared when the PEI/dsRNA-p21 ratio in weight was 2 whether with or without adding HA, indicating the disassembly of ternary complexes did not occur. However, signal of dsRNA-p21 was detectable in the well for ternary complexes, showing the loosening of PEI/dsRNA-p21 complexes. Some research reported that, the addition of poly-anion might lead to competitive dissociation of RNA from the poly-cation (Arigita et al., 1999; Koyama et al., 2002). However, our results showed that all of the studied TC- dsRNA-p21 with HA/PEI weight ratios from 1 to 8 were retarded in the loading wells, clearly indicating that the anionic shielding could not decompose the PEI/dsRNA-p21 complexes. We assumed that, the HA only shield the surface of PEI/dsRNA-p21, loosening the out layer of binary complexes, and the negative charges of HA was not strong enough to dissociate RNA from BC-dsRNA-322. This result agrees with previous research (Wang et al., 2011; Park et al., 2016).

Nucleic acids especially RNAs are often unstable in physiological conditions and degraded by nucleases (Chen et al., 2016). Thus, it is necessary to evaluate the protective effect of different carriers todsRNA-p21. In this study, potent RNase A was used to investigate the integrity of the dsRNA-p21 encapsulated in the binary complexes and ternary complexes with a variety of HA concentrations. Naked dsRNA-p21 was used as control. Samples were incubated with RNase A for 1.5 h, and the degradation of dsRNA-p21 by RNase A was evidenced by the faint smear of dsRNA-p21 down the lane. As shown in Figure 2(B), a complete degradation was observed for the naked dsRNA-p21, with no detectable signals for the intact dsRNA-p21. The dsRNA-p21 in binary complexes was well protected against RNase A. The migration position of the dsRNA-p21 band indicated that RNA degradation under RNase treatment was less than that of free dsRNA-p21. The dsRNA-p21 in ternary complexes was more resistant to the treatment with RNase, with bright signal of dsRNA-p21 brand almost similar to that of the control one, when the HA/PEI was above 4. The enhanced protective activity of the ternary complexes might attribute to the combined effect of PEI and HA. Size change of the binary and ternary complexes in physiological salt condition was determined by DLS. As shown in Figure 2(C), the PEI/dsRNA-p21 complexes were unstable and aggregated quickly. It even reached a diameter of 1.2 μm after 1 h incubation in physiological salt condition. It might be explained by the reason that the salt ions in the solution displaced hydrogen bounded to the amino group, reduced the repulsive barriers between PEI/dsRNA-p21 particles, leading to the aggregating of particles *via* inter-particle cross-bridging (Trubetskoy et al., 1999). Comparing to the PEI/dsRNA-p21 complexes, the stability of TC-dsRNA-p21 was dramatically improved. When the HA/PEI ratio reached to 6, the HA/PEI/dsRNA-p21 ternary complexes remained about 286.7 ± 16.3 nm after 6 h incubation in physiological salt condition. As the HA contained negative charge, the HA shell could absorb water through the hydrogen–bond interaction. It was probably due to the strong repulsive strength between negative-charged particles, combined with hydration shell made the ternary complexes displayed excellent stability in physiological condition.

The resistance to RNase, stability in physiology condition and optimal RNA condensation capability of TC-dsRNA-p21 might make an effective dsRNA-p21 delivery possible *in vivo*.

### 3.2. Cytotoxicity evaluation

The cytotoxicity of the materials in the Lovo and 293T cells was evaluated using the MTT assay. As showed in Figure 2(D and E), cytotoxicity was significantly increased at high concentration of dsRNA-p21 for both cell lines. The reason could be the strong positive charge of the PEI/dsRNA-p21 binary complexes that compromised the integrity of the cell membrane and lead to significant cell death (Fischer et al., 2003; Moghimi et al., 2005). For BC-dsRNA-p21, the cell viability dropped to under 80% for Lovo cells and 70% for 293T cells, when the dsRNA-p21 concentration was above 25 μg/ml. When the dsRNA-p21 concentration was 100 μg/ml, the cell viability further plummeted under 30% and 40%, respectively. However, the cytotoxicity of TC- dsRNA-p21 was significantly decreased with the addition of HA. As nature extracellular matrix, HA showed high biocompatibility. The viabilities of both Lovo and 293T cells treated with TC- dsRNA-p21were80% after 24 h exposure, even when the dsRNA-p21 concentration went up to 100 μg/ml and the HA/PEI weight ratio was above 4. These results confirmed the super bio-compatibility of TC- dsRNA-p21 delivery system.

### 3.3. Intracellular uptake

**Figure 3.**
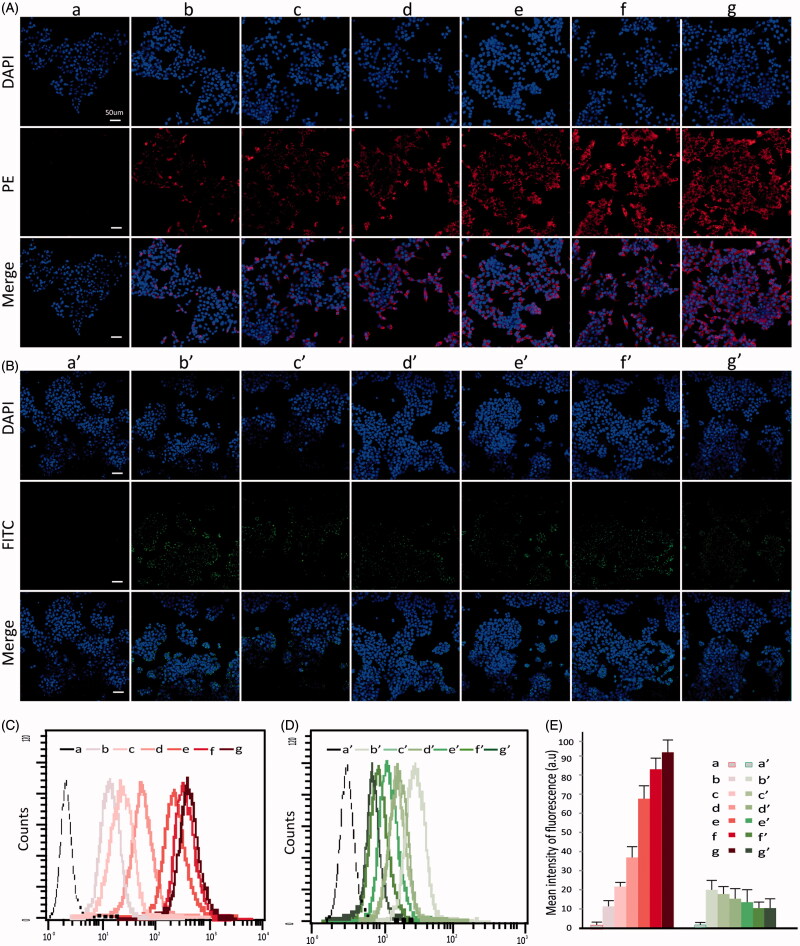
The Lovo and 293 T cell uptake efficiency after 6 h incubation with FITC-dsRNA-p21 or PE-dsRNA-p21 encapsulated systems. (A) Lovo cells were incubated with PE-dsRNA-p21 containing formulations at the dsRNA-p21 concentration of 50 nM for 6 h. (B) 293 T cells were incubated with FITC-dsRNA-p21 containing formulations at the dsRNA-p21 concentration of 50 nM for 6 h. *In vitro* cellular uptake was analyzed using CLSM. Blue DAPI dye stained the cell nucleus, the fluorescence of PE was red and the fluorescence of FITC was green. The multiple-exposure image of CLSM was acquired with filter (emission maximum 439 nm for DAPI, 680 nm for PE and 530 nm for FITC). (C) *In vitro* cellular uptake of Lovo cells was evaluated using flow cytometric analysis. (D) *In vitro* cellular uptake of 293 T cells was evaluated using flow cytometric analysis. (E) Mean intensity of fluorescence. Excitation was by a single 15 mW argon-ion laser beam (488 nm). Emission was collected through a 530 nm band for FITC and 680 nm for PE. a–g represents untreated, PEI/dsRNA-p21 at weight ratio of 1:1, HA/PEI/dsRNA-p21/at HA/PEI weight ratio of 1, HA/PEI/dsRNA-p21/at HA/PEI weight ratio of 2, HA/PEI/dsRNA-p21/at HA/PEI weight ratio of 4, HA/PEI/dsRNA-p21/at HA/PEI weight ratio of 6 and HA/PEI/dsRNA-p21/at HA/PEI weight ratio of 8 treated Lovo cells. a’-g’ represents untreated, PEI/dsRNA-p21 at weight ratio of 1:1, HA/PEI/dsRNA-p21/at HA/PEI weight ratio of 1, HA/PEI/dsRNA-p21/at HA/PEI weight ratio of 2, HA/PEI/dsRNA-p21/at HA/PEI weight ratio of 4, HA/PEI/dsRNA-p21/at HA/PEI weight ratio of 6 and HA/PEI/dsRNA-p21/at HA/PEI weight ratio of 8 treated 293 T cells. Error bars represent means ± SD for *n* = 3.

To learn the cellular uptake, the FITC-as well as PE-tagged dsRNA-p21 was entrapped in different formulations and incubated with 293TT cells or Lovo cells for 6 h. The uptake was monitored using confocal laser scanning microscope (CLSM) and flow cytometry. As shown in Figure 3, the free dsRNA-p21 was not readily taken up by the cells. The BC-dsRNA-p21 also demonstrated low uptake rate, which agreed with previous research (Xu et al., 2009). The instability in serum-containing medium and easy to aggregation might be the main reason of low transfection efficacy of the BC-dsRNA-p21. The uptake of TC-dsRNA-p21 showed a difference between 293TT and Lovo cells. As shown in Figure 3(A and B), the TC-dsRNA-p21 demonstrated lower uptake by the 293TT cells but higher one by the Lovo cells, with respecttoBC-dsRNA-p21 did. The TC-dsRNA-p21 with a HA/PEI ratio at 8 demonstrated the highest uptake in the Lovo cells. The cellular uptake of TC-dsRNA-p21 with HA/PEI ratio above 6 was visually higher than that of BC-dsRNA-p21 and TC-dsRNA-p21 with a HA/PEI ratio below 4. However, in the 293 T cells, the cellular uptake of TC-dsRNA-p21 showed slightly lower than that of BC-dsRNA-p21. The results came from flow cytometric analysis was inconsistent with that from CLSM (Figure 3(C and D)). The cellular uptake of TC-dsRNA-p21 with HA/PEI ratio above 6 was significantly higher than that of BC-dsRNA-p21 and TC-dsRNA-p21 with a HA/PEI ratio below 4, and there was no significant difference for the uptake of TC-dsRNA-p21 when the PEI/HA ratio was between 6 and 8 in Lovo cells. In the 293T cells, the difference of cellular uptake was not significant among all the studied dsRNA-p21 containing systems. With the negative HA shielding, the interaction of ternary complexes with negatively charged cell membrane was restricted. The adsorptive endocytosis might be suppressed in the293TT cells. However, for CD44 abundant cells, the cellular uptake of TC-dsRNA-p21 might be increased *via* HA-CD44 mediated endocytosis. The difference of uptake efficiency between 293T cells and Lovo cells proved that, after being shielded with HA, the ligand-receptor mediated endocytosis was the main internalization mechanism for TC-dsRNA-322. CD44, the specific receptor of HA, was highly expressed in Lovo cells leading to the increased uptake of TC-dsRNA-322. We assume that the PEI/nucleic acid complexes were up-taken by the cells through a physical reaction (positive vs negative) (Godbey et al., 1999; Whitehead et al., 2009), which mainly represents a surface contact between the two subjects. While HA modulated ternary complexes is taken by the cancer cells through a ligand-receptor mediated interaction (HA vs CD44), which covers either surface contact or biological uptake process, leading to a high level of uptake.

### 3.4. Activating effect on p21 by the dsRNA-p21-containing systems

**Figure 4.**
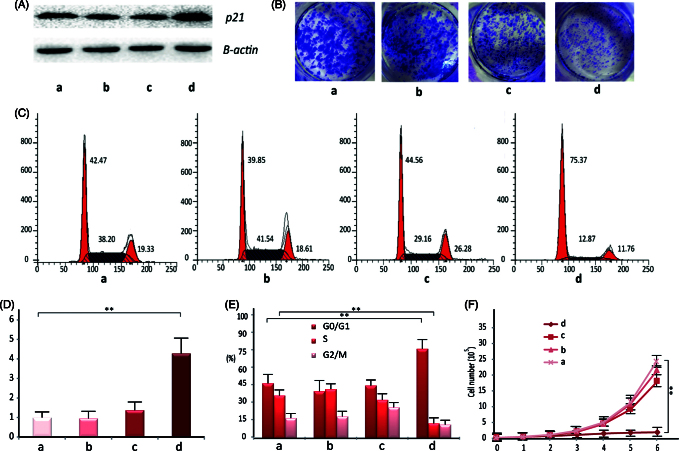
The *in vitro* pharmacological effect of different dsRNA-p21 containing formulations on Lovo cells. Lovo cells were transfected with 50 nM of the indicated dsRNA-p21 containing BC (c) and TC (d) for 48 h, untreated (a) and naked dsRNA-p21 (b) were used as control. (A) Induction of P21 protein expression was detected by Western blotting analysis. The results were normalized to b-actin as density ratio. (B) Colony formation analysis. (C) Activation of p21 by TC-dsRNA-p21 caused cell cycle arrest of Lovo cells at *G*_1_/*G*_0_. (D) Induction of p21 mRNA expression was analyzed by RT-PCR. The results were presented as means ± SD of three independent experiments and normalized to GAPDH. Expression levels were measured as fold relative to that of mock group. (E) Shown is the representative graph indicating cell distribution in the *G*_1_/*G*_0_, *G*_2_/M and S phases. (F) The TC-dsRNA-p21suppressed cell proliferation in Lovo colorectal cancer cells. Cell proliferation was determined by cell counting on a daily basis. Each time point data represents the mean ± standard deviation of six independent experiments. Cells of reference groups showed an exponential growth, whereas the growth of the cells with TC-dsRNA-p21 transfection was markedly suppressed. (**p < .01 and ***p < .005 compared to untreated group).

Lovo cells were chosen for transfection efficiency studies. The BC-dsRNA-p21 at PEI/dsRNA-p21 weight ratio of 2 and TC-dsRNA-p21at HA/PEI ratio of 6 were prepared, respectively, at pH value of 7.4 and NaCl concentration of 20 mM. The dsRNA-p21 water solution and untreated cells were as control. The *in vitro* transfection experiment was performed in the presence of 10% fetal calf serum. Western blotting and RT-PCR method was used to detect the expression of p21 in the Lovo cells 48 h after transfection. As seen in Figure 4(A and D), the transfection efficiency in Lovo cells was significantly improved with TC- dsRNA-p21, as compared to that withBC-dsRNA-p21. It might be interpreted by the improved cellular uptake efficiency and dsRNA-p21 dissociation inside the cells. Furthermore, the HA shell loosened the electrostatic complexation between PEI and dsRNA-p21, which might promote RNA liberation inside the cells.

### 3.5. *In vitro* pharmacological effect

P21 is particularly important in the development of CRC. Numerous results demonstrated that, p21 plays a dominant role in regulating cell cycle (Kalimutho et al., 2011), suspending cell proliferation at *G*_0_/*G*_1_ phase (Wang et al., 2010) and suppressing colony formation (Wang et al., 2017) in human CRC. Thus, we tested the *in vitro* pharmacological effect of TC-dsRNA-p21 on the Lovo cells following activation of p21 expression. As shown in Figure 4(C and E), percentage of the cells in the *G*_0_/*G*_1_ phase was significantly elevated in the TC-dsRNA-p21 treated group (75.37%), as compared to that in the BC-dsRNA-p21 (44.56%), mock (39.85%) and untreated group (46.47%). Treatment with TC-dsRNA-p21 also decreased the number of cells in S phase (12.87%), in respect to those transfected with the BC-dsRNA-p21 (29.16%), mock (41.54%) and untreated (36.20%) ones, confirming the TC-dsRNA-p21caused cell cycle arrest at the *G*_0_/*G*_1_ checkpoint. At the same time, the growth and colony formation of cancer cells were dramatically suppressed by TC-dsRNA-p21. As shown in Figure 4(F), the growth platform of the Lovo cells was observed two days after incubating with TC-dsRNA-p21, while cells treated with BC-dsRNA-p21, or mock, or culture medium (untreated) showed an exponential growth. The antitumor efficacy was also verified with colony formation. As shown in Figure 4(B), the cells transfected with TC-dsRNA-p21 showed significantly lower colony formation, as compared with reference groups. The results of the *in vitro* pharmacological effect proved potential of the TC-dsRNA-p21 for treatment of CRC via activating tumor suppressor genes.

### 3.6. *In vivo* retention and bio-distribution

**Figure 5.**
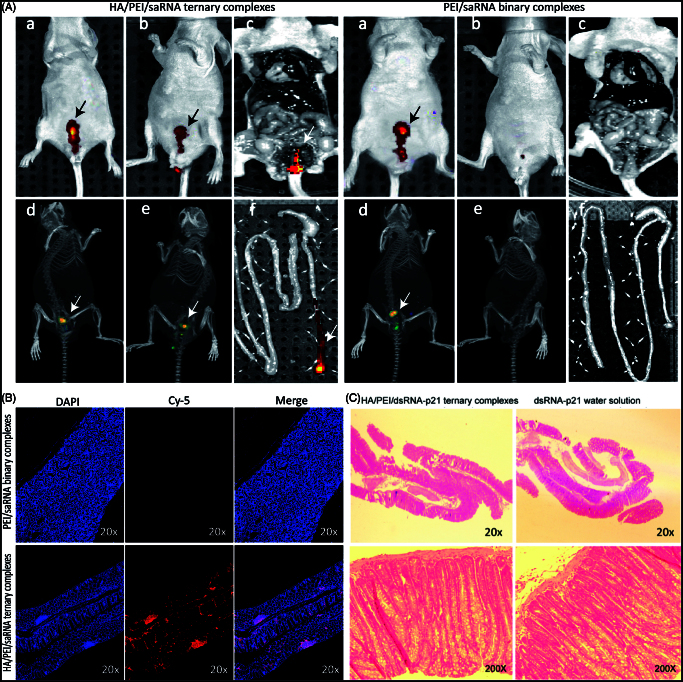
Bio-distribution and rectal irritation assay. The cy5-dsRNA-p21 entrapped TC ternary was administered into BALB/c mice via rectal injection. The cy5-dsRNA-p21 water solution was used as control. About 5 min and 3 h after treatment, fluorescent signals were collected using IVIS Spectrum Imaging System. 10 h later, mice were sacrificed and the colorectal tissue was carefully collected for frozen section and counter-stained with DAPI as well as HE stain. (A–a) and (A–d). Represent 2 D and 3 D fluorescent imaging, respectively 5 min after treatment. (A–b) and (A–e). Represent 2 D and 3 D fluorescent imaging, respectively 3 h after treatment. (A–c). Represent 2 D fluorescent imaging of postmortem. (A–f). Represent fluorescent imaging of colorectal tissue. (B) Samples were visualized by fluorescent microscopy. Representative images are cy5-p21-saRNA-322 uptake (red) and nuclear staining (DAPI, blue) in colorectal wall. (C) The morphology of rectal tissues after exposure to TC-dsRNA-p21 and dsRNA-p21 water solution.

In the present study, fluorescent imaging (FRI) were applied to assess the bio-distribution of *in situ* applied TC-dsRNA-p21. The cy-5conjugated dsRNA-p21 was formulated in the ternary system, and the nude mice were treated with the TC-cy5-dsRNA-p21 via rectal administration, followed by monitoring fluorescent signal in a noninvasive fashion. The cy5-dsRNA-p21 water solution served as control. As depicted in Figure 5(A), the difference in colorectal retention between the test group and control group was significantly visualized. Five minutes after treatment, both groups showed considerable fluorescent signal in the rectal site (Figure 5(Aa and Ba)). Tomography configuration of the fluorescent signal (Figure 5(Ad and Bd)) proved the localization, implying a successful delivery of the drug to the target site. About 3 h post treatment, the fluorescent signal entirely faded away in the animals treated with cy5-dsRNA-p21 water solution, while TC-dsRNA-p21 treated animals kept strong fluorescent signals at the applied site (Figure 5(Ab and 5Bb)). About 10 h after administration, animals were sacrificed and the colon tissues were taken. The frozen sections were made followed by counter-stained with DAPI. The Fluorescent signal of cy5-dsRNA-p21 was still visualized in the postmortem animals in the TC-cy5-dsRNA-p21 treated group, while no signal was detected in the animals of control group (Figure 5(Ac, Bc, Af and Bf)). It’s noteworthy that, the strong signals were accumulated at colorectal site, with no detective signal in the surrounding tissue. This might be beneficial for improving therapeutic effect, as well as reducing the unexpected side effect. The distribution of cy5-dsRNA-p21 in the colorectal wall was also detected under fluorescent microscopy (Carl Zeiss, Germany). As exhibited in Figure 5(B), in the colorectal wall, the fluorescent signal in the TC-dsRNA-p21 group was stronger than that in the control group, providing a clear evidence for successful accumulation of therapeutic agents on target site. The triumphant collection of the active reagent on target site might be merited from the bio-adhesive property of HA, which enhanced drug contact to the mucosal surface of the colorectal wall.

### 3.7. Rectal irritation

Morphological test showed that the TC-dsRNA-p21 caused no irritation or damage on rectal tissues during the treatment course (Figure 5(C)). These improvements were likely due to the biocompatibility of the HA shell, which minimized the direct contact of PEI to the rectal mucous.

## 4. Conclusions

In this study, a ternary system consisting of PEI/RNA binary core and HA corona was developed. The introduction of HA increased the stability, reduced myotoxicity and enhanced the cellular uptake in the Lovo cells due to HA-CD44 mediated endocytosis. *In vitro* pharmacodynamics test showed that, the TC-dsRNA-p21 significantly increased p21 expression in Lovo cells, which in turn generated inhibitory effect on cancer cell growth. *In vivo* distribution experiment showed that the system accumulated drugs in the colorectal site. The small active RNA (saRNA) induced gene expression represents a novel approach for the treatment of human cancer. However, the RNAa-based clinical research has been restricted due to the lack of efficient delivery systems. The delivery system in the present study might be a useful tool to achieve this aim and worth further exploration. In whole, this system might have potential for the treatment of CRC.
